# Unmet Healthcare Needs in COPD: A Text Network Analysis and Topic Modeling of Pre/Post-COVID-19 Research Trends

**DOI:** 10.3390/healthcare14010082

**Published:** 2025-12-29

**Authors:** So Young Yun, Mi Ok Song

**Affiliations:** 1 Department of Nursing, Nambu University, Gwangju 62271, Republic of Korea; yunsy@nambu.ac.kr; 2 Department of Nursing, Mokpo National University, Muan 58554, Republic of Korea

**Keywords:** chronic obstructive pulmonary disease, health services needs and demand, healthcare access, health disparities

## Abstract

**Highlights:**

**What are the main findings?**

- Research on COPD increasingly recognizes that patients face barriers not only in treatment but also in diagnosis, rehabilitation, and access to medicines.
- Since COVID-19, studies have shifted toward digital tools, remote support, and fairer access to medication and services.

**What are the implications of the main findings?**

- Improving care for people with COPD means building health systems that see the whole person—addressing medical needs alongside access, affordability, and support.
- Expanding digital care options, strengthening rehabilitation programs, and ensuring fair access to treatment can help people with COPD live healthier, more stable lives.

**Abstract:**

**Background/Objectives:** Unmet healthcare needs, driven by structural and patient-level barriers, are particularly critical in chronic obstructive pulmonary disease (COPD). However, limited research has examined how academic themes on this topic connect and evolve over time. This study analyzed the structure and temporal shifts in research trends on unmet healthcare needs in COPD to identify key concepts and topics and policy implications. **Methods:** We systematically searched PubMed, Embase, and CINAHL (12–15 March 2025) to identify English-language abstracts on unmet healthcare needs in COPD. Eligible studies were peer-reviewed articles with an English-language abstract that examined unmet healthcare needs from the patient perspective. In total, 451 abstracts were analyzed using text network analysis and Latent Dirichlet Allocation. Topic distributions before and after the coronavirus disease pandemic were assessed using chi-square tests, and findings were interpreted within Penchansky and Thomas’s 5A healthcare access framework. **Results:** Six topics emerged: socioeconomic disparities, early diagnosis and symptom management, guideline-based information and technology use, integrated care for advanced COPD, access to pulmonary rehabilitation, and equitable medication availability. These topics mapped onto all five access dimensions, underscoring the multidimensional nature of unmet healthcare needs. Network analysis identified management, diagnosis, symptoms, exacerbation, and other related terms as central hubs in the discourse. Post-pandemic, research shifted toward digital information delivery, technology adoption, and equitable pharmacotherapy. **Conclusions:** Findings suggest that reducing unmet healthcare needs in COPD requires integrated systems that address both disease complexity and access barriers. Targeted, multidisciplinary, and policy-driven interventions in highly central domains are needed to reduce disparities and improve outcomes. This study also confirmed a post-pandemic shift in research priorities, emphasizing the need for equitable and adaptive healthcare policies.

## 1. Introduction

Unmet healthcare needs arise when individuals perceive or experience a need for medical care but are unable to obtain adequate and timely services due to barriers such as cost, limited access, or insufficient information [1]. The concept extends beyond delayed treatment, encompassing implications for disease prevention, early intervention, and overall quality of life. Accordingly, unmet healthcare needs serve as critical indicators of healthcare access [2] and are increasingly recognized as key metrics for monitoring progress toward Universal Health Coverage (UHC). They are particularly salient in relation to the health rights of vulnerable populations and individuals with chronic conditions [1]. Unmet healthcare needs reflect the interplay of individual determinants such as health perceptions and health literacy and structural barriers such as economic constraints, regional disparities, and service quality, which may be further compounded by institutional and policy constraints [1,3].

Chronic obstructive pulmonary disease (COPD) is a progressive respiratory condition that requires long-term, multifaceted management, including self-care, regular clinical follow-ups, preventive interventions, and rehabilitation therapy [4,5]. However, many patients encounter barriers such as financial hardship, mobility limitations, insufficient availability of medical services, and limited disease awareness. These barriers undermine treatment adherence, accelerate disease progression, and increase the risk of hospitalization [6]. Calverley [7] categorized the unmet healthcare needs of patients with COPD into patient-related and provider-related domains. Patient-related needs included more effective diagnosis, improved symptom control, management of disease progression, and enhanced survival. Similarly, Clari et al. [8] reported that patients frequently experienced unmet healthcare needs related to information provision, emotional support, and social care. In particular, healthcare access, delays in diagnosis and treatment, nonadherence to clinical guidelines, and limited availability of palliative care continue to be identified as unmet needs [9,10].

However, most studies have examined these issues in isolation, and few have investigated how diverse research topics on unmet healthcare needs in COPD are interconnected within the academic discourse. To systematically conceptualize these barriers, Penchansky and Thomas [11] proposed five dimensions of healthcare access: availability, accessibility, affordability, acceptability, and accommodation. This framework has been widely applied to examine structural inequities in healthcare delivery. It encompasses not only the availability of medical resources but also patients’ ability to access, afford, and accept them within a responsive healthcare system.

Following the World Health Organization’s declaration of coronavirus disease (COVID-19) as a global pandemic in March 2020, healthcare utilization among vulnerable populations, including individuals with COPD, was substantially disrupted. COPD is a major underlying condition associated with a heightened risk of severe illness and mortality from COVID-19 [12]. Numerous studies have reported marked declines in outpatient visits and hospital admissions for patients with COPD during the pandemic [13,14], suggesting that these disruptions may have precipitated structural shifts in healthcare access and delivery systems. Consequently, the thematic focus and discourse patterns related to unmet healthcare needs in COPD have likely also evolved.

Despite these developments, few longitudinal analyses have examined research trends on unmet healthcare needs in COPD, particularly with respect to changes in discourse structures before and after the COVID-19 pandemic. Moreover, it remains unclear how the knowledge structure and thematic emphases of this literature have evolved over time. To address this gap, this study aimed to identify the types of unmet healthcare needs reported among patients with COPD in the academic literature, examine key concepts related to these needs using keyword network analysis, and analyze how research topics on unmet healthcare needs in COPD have shifted before and after the COVID-19 pandemic. To achieve these aims, the present study applied text network analysis (TNA) [15] and topic modeling to academic literature and interpreted the findings within the 5A framework of healthcare access. The results are expected to provide evidence to inform patient-centered health policies and service designs that aim to reduce disparities in this population.

## 2. Materials and Methods

### 2.1. Study Design

This study employed a bibliometric approach, combining systematic literature retrieval with computational text mining methods. Specifically, TNA and topic modeling were applied to the abstracts of academic publications on unmet healthcare needs in patients with COPD. Key terms were extracted from the abstracts to construct a keyword co-occurrence network and identify temporal changes in research topics.

### 2.2. Literature Search

A systematic search was conducted in three electronic databases—PubMed, Embase, and CINAHL—between 12 and 15 March 2025. The selected databases are widely used in health and nursing research and were chosen to capture a broad range of clinical, public health, and patient-centered studies relevant to the research question. Search terms were developed iteratively based on the core concepts of the research question and a preliminary scoping search. The search strategy combined Medical Subject Headings (MeSH) with free-text terms for COPD (e.g., “Chronic Obstructive Pulmonary Disease,” “Chronic Bronchitis,” “Emphysema”) and multiple variants of unmet healthcare needs (e.g., “unmet health needs,” “healthcare gaps,” “healthcare service deficiency”). Boolean operators (AND, OR) were used to combine the search terms. Additional terms related to healthcare accessibility and disparities were also included. The strategy was tailored for each database, and detailed search strings are provided in Appendix A. No restrictions were imposed on publication year, study design, or country of origin.

### 2.3. Eligibility Criteria

Studies were eligible for inclusion if they met the following criteria: (1) publication in peer-reviewed journals, (2) availability of an English-language abstract, and (3) examination of unmet healthcare needs from the patient perspective. Only English-language abstracts were included to ensure linguistic consistency for text-based analyses, including keyword extraction, topic modeling, and the development of dictionaries for synonyms, exclusion terms, and designated terms in text network analysis. The patient perspective was defined as abstracts explicitly reporting patient-experienced barriers, perceived difficulties in accessing care, or patient-reported unmet needs. This included studies addressing patient experiences, perceptions, satisfaction, or barriers to healthcare utilization, as well as patient-reported outcomes (e.g., quality of life, symptom burden) used to discuss access-related gaps or unmet needs. During abstract screening, records were retained only when the abstract explicitly indicated patient-level evidence on needs, barriers, or access gaps.

Studies were excluded if they (1) lacked an abstract or were not written in English, (2) were not original research articles (e.g., editorials, commentaries, letters, conference papers, or workshop reports), (3) did not focus on COPD, or (4) addressed unmet healthcare needs solely from the perspective of policymakers or healthcare providers. In addition, studies focusing on system- or policy-level aspects-such as cost prediction, epidemiological trends in prevalence or mortality, treatment innovations, or policy implementation-were excluded. This approach reflects the observation by Vreman et al. [3] that unmet healthcare needs can be interpreted differently by various stakeholders, including medicine developers, regulators, payers, and health technology assessment bodies. Accordingly, the present study focused specifically on the patient-centered dimension of unmet healthcare needs among these multiple interpretations.

### 2.4. Study Selection

**Figure 1:**
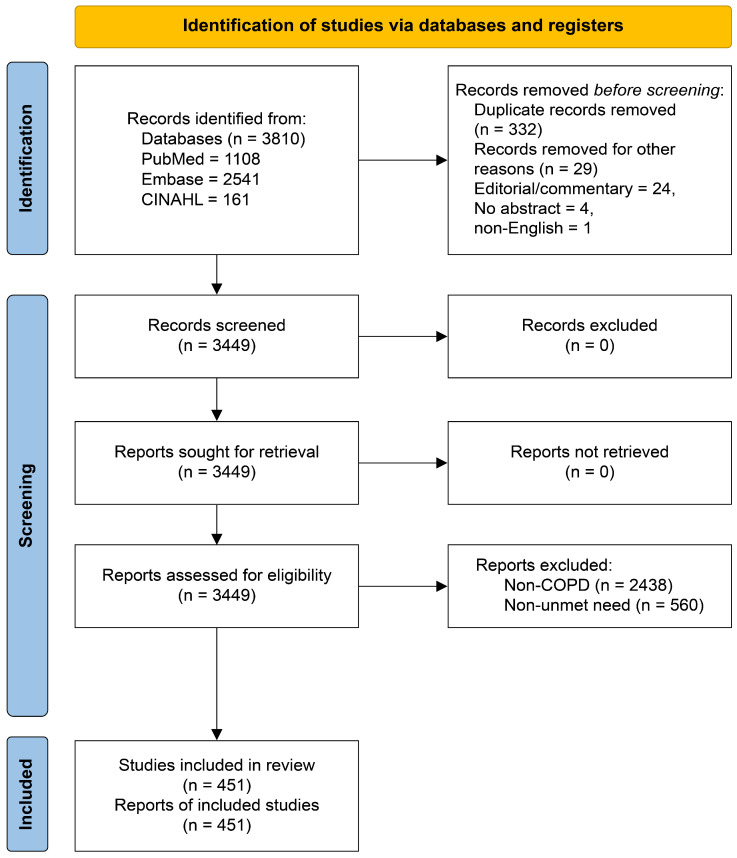
Study selection process following the PRISMA 2020 flow diagram guidelines [16].

The initial search retrieved 3810 records (PubMed, 1108; Embase, 2541; CINAHL, 161). After removing duplicate records (n = 332) and records removed for other reasons (n = 29; editorials/commentaries, n = 24; no abstract, n = 4; non-English, n = 1), 3449 records remained for screening. Two researchers independently screened all titles and abstracts against the eligibility criteria. When there was a discrepancy, we discussed it to reach an agreement by consensus. During abstract-level eligibility assessment, records were excluded primarily because they did not focus on COPD (n = 2438) or did not address unmet healthcare needs (n = 560), resulting in 451 articles retained for the final analysis. The literature identification and study selection process was conducted and reported in accordance with the Preferred Reporting Items for Systematic Reviews and Meta-Analyses (PRISMA) 2020 statement to enhance transparency and reproducibility of the staged screening and exclusion process. The study selection process is illustrated in the PRISMA flow diagram (Figure 1).

### 2.5. Data Preprocessing

The selected publications were converted into an analyzable format using NetMiner 4.4 (Cyram Inc., Seongnam, Republic of Korea) [17]. Morphological analysis was conducted using NetMiner’s cleaning and extraction functions, and only nouns were extracted from the abstracts. To enhance semantic clarity, three custom dictionaries were constructed: synonym, stopword, and compound-term dictionaries (examples are provided in Appendix A; processed metadata and topic modeling outputs used for analysis are available in Supplementary Materials File S1 Dataset).

All extraction and refinement procedures were independently reviewed and finalized through discussion by two researchers, one of whom had expertise in text mining. This expertise contributed to ensuring the validity, reliability, and rigor of the preprocessing process. Synonymous or semantically similar terms were consolidated into a single representative term, and irrelevant or redundant terms were removed during data refinement.

### 2.6. Generation of Keyword Network

Following preprocessing, the top 30 high-frequency keywords were selected for analysis, based on their frequency of occurrence across article abstracts. The top 30 keywords were selected for analysis to ensure a balance between capturing the core thematic structure of the literature and maintaining analytical clarity. This threshold has been commonly used in previous keyword-based content and network analyses to enhance interpretability while avoiding excessive sparsity [18,19]. A two-mode network (articles × keywords) was converted into a one-mode network (keywords × keywords) to evaluate keyword co-occurrence. To reduce redundancy and enhance interpretability, the PathFinder Network (PFNet) algorithm was applied, retaining the most meaningful, high-weight connections [20]. Three centrality measures—degree, betweenness, and closeness—were computed to assess the relative importance of keywords within the network [21].

### 2.7. Topic Modeling

Latent Dirichlet Allocation (LDA) was conducted using the “Evaluation of Topic Models” function in NetMiner. LDA is a generative statistical model, describing observations through unobserved groups, explaining similarities in parts of the data [22]. LDA enables the identification of hidden topics in the analyzed research, overall dataset topics, and the proportion of topics in each literature [22]. During the simulation, five to ten topics were explored, with the alpha (α) parameter ranging from 0.1 to 0.01, beta (β) fixed at 0.01 [23], and Gibbs sampling performed with 1000 iterations to ensure convergence. Model selection was guided by topic coherence [24], specifically the cv and UMass metrics, which assess the consistency of semantically related words. For each finalized topic, the top keywords were extracted, and the titles of associated articles were reviewed. Interpretive topic labels were then collaboratively assigned by the research team. Finally, to strengthen theoretical interpretability, the six topics were mapped onto the five dimensions of healthcare access proposed by Penchansky and Thomas [11]—availability, accessibility, affordability, acceptability, and accommodation—through team discussion and consensus. To ensure rigor, the mapping results were validated through independent expert review by a professor specializing in public health.

### 2.8. Topic Trend Analysis Before and After COVID-19

To examine the impact of the COVID-19 pandemic on research themes and keyword emergence related to unmet healthcare needs in COPD, the study period was divided into two phases: pre-pandemic (before 2020) and post-pandemic (2020–2025). The WHO’s official declaration of the pandemic in March 2020 was used as the reference point [25].

Statistical analyses were conducted to examine differences in topic distributions across the pre- and post-pandemic periods. A chi-square test of independence was applied to assess whether the proportion of topics differed significantly between the two phases. Statistical significance was determined at a two-tailed α level of 0.05. All analyses were performed using SPSS version 26.0 (IBM Corp., Armonk, NY, USA).

### 2.9. Ethics Statement

This study did not involve human participants and therefore did not require Institutional Review Board (IRB) approval or informed consent. All data analyzed were obtained from publicly available literature databases (PubMed, Embase, and CINAHL), and no individual-level or identifiable information was used.

This section may be divided by subheadings. It should provide a concise and precise description of the experimental results, their interpretation, as well as the experimental conclusions that can be drawn.

## 3. Results

### 3.1. Article Selection and Temporal Distribution

A total of 451 articles were included in the final analysis. The earliest study was published in 1998, and the number of publications has generally increased over time. By year range, 53 articles (11.7%) were published between 1998 and 2009, 202 (44.8%) between 2010 and 2019, and 196 (43.5%) during the post-pandemic period (2020–2025).

### 3.2. Keyword Network Analysis

From the abstracts of 451 articles, 4382 keywords were extracted after preprocessing. Among the top 30 extracted keywords, the most frequently occurring terms were management (133 articles), diagnosis (114), symptom (111), exacerbation (110), and palliative care (102). In terms of degree centrality, management (0.172) and symptom (0.172) ranked highest, followed by exacerbation (0.138), palliative care (0.138), and spirometry (0.138). For betweenness centrality, symptom (0.700) and exacerbation (0.628) showed the strongest bridging roles, with management (0.370), palliative care (0.315), and diagnosis (0.296) also exhibiting high values. Closeness centrality results similarly highlighted symptom (0.377), exacerbation (0.367), management (0.305), diagnosis (0.305), and palliative care (0.305) as the most central nodes in the network (Table 1). Application of the PathFinder Network (PFNet) algorithm reduced the initial 434 co-occurrence linkages to 29 meaningful, high-weight connections, thereby reducing redundancy and enhancing the interpretability of the network. In this pruned structure, management, diagnosis, symptom, exacerbation, palliative care, pulmonary rehabilitation, and hospitalization appeared frequently and were connected with multiple keywords.

**Figure 2:**
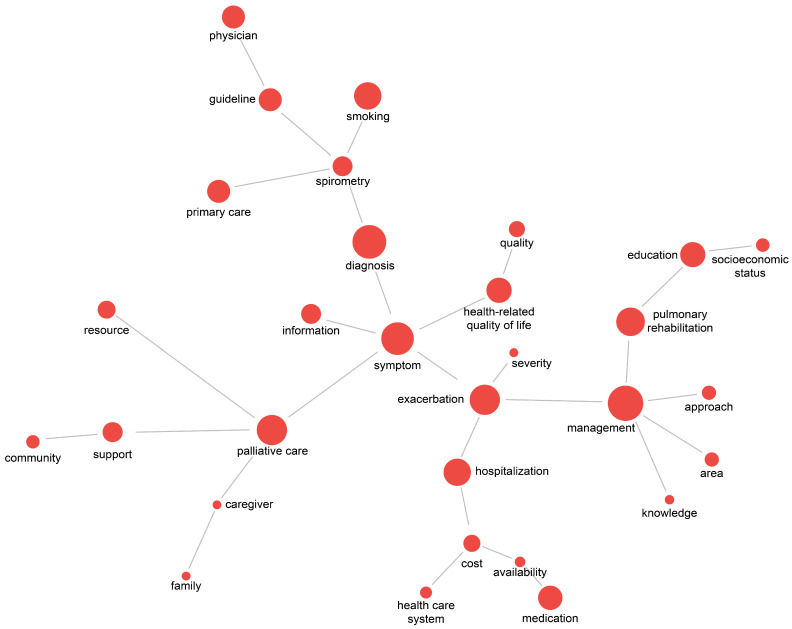
Keyword co-occurrence network of unmet healthcare needs in COPD.

The spatial distribution of keywords indicated distinct research domains. On the right side of the network, studies related to management and education, including pulmonary rehabilitation, were positioned. At the center, symptom and exacerbation clustered together, reflecting research on symptom management and acute exacerbations. At the bottom, exacerbation was linked with hospitalization, forming a cluster related to admission and cost and other related factors. On the left, palliative care was associated with caregiver, family, and support, representing studies on palliative and supportive care. At the top, diagnosis was connected with spirometry and smoking, among others, indicating research on diagnosis and early detection. This structure illustrates that research on unmet healthcare needs in COPD is organized into topic-specific clusters (Figure 2).

### 3.3. Topic Modeling of Unmet Healthcare Needs in COPD

The optimal LDA model (α = 0.04, β = 0.01, c_v = 0.603, UMass = −2.033) identified six topics. Topic 1 represented socioeconomic inequalities in COPD outcomes; Topic 2, early diagnosis and symptom management; Topic 3, guideline-based information and technology use; Topic 4, integrated care for advanced COPD; Topic 5, improving access to pulmonary rehabilitation; and Topic 6, availability and equity of COPD medications.

For each topic, the top seven keywords were extracted, and the titles of associated articles were reviewed. Based on these results, the research team collaboratively assigned interpretive labels to each topic group (Table 2). The six topics were then mapped onto the five healthcare access dimensions proposed by Penchansky and Thomas [11]. Topic 1 corresponded to availability, accessibility, and affordability; Topic 2, availability and accessibility; Topic 3, acceptability and accommodation; Topic 4, availability, accommodation, and acceptability; Topic 5, availability, accessibility, accommodation, and affordability; and Topic 6, availability and affordability. The detailed keywords supporting each classification are provided in Table 2.

### 3.4. Topic Trends Before and After the COVID-19 Pandemic

**Figure 3:**
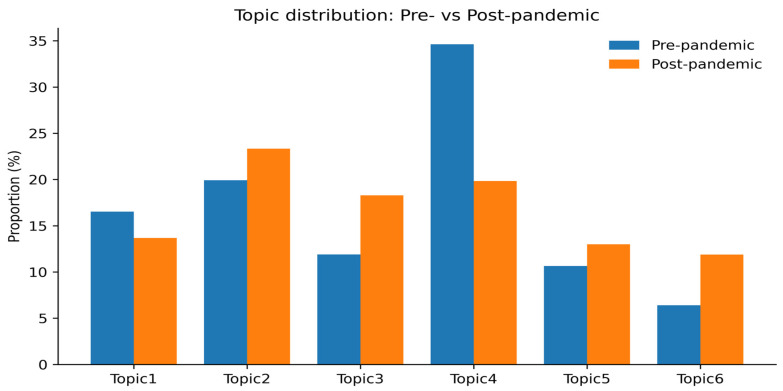
Comparison of topic proportions in COPD research on unmet healthcare needs: pre- and post-COVID-19.

Before the pandemic, Topic 4 (integrated care for advanced COPD) accounted for the largest share of the discourse, representing 32.2% (82 articles); however, this proportion declined to 20.4% (40 articles) after the pandemic. In contrast, the proportion of articles assigned to Topic 3 (guideline-based information and technology use) increased from 13.3% (34 articles) to 19.4% (38 articles), and Topic 6 (availability and equity of COPD medications) rose from 6.7% (17 articles) to 11.7% (23 articles). Topic 2 (early diagnosis and symptom management) showed a slight increase from 19.6% (50 articles) to 21.4% (42 articles), while Topic 5 (improving access to pulmonary rehabilitation) increased from 12.6% (32 articles) to 14.3% (28 articles). In contrast, Topic 1 (socioeconomic inequalities in COPD outcomes) decreased from 15.7% (40 articles) to 12.8% (25 articles). Overall, the distribution of topics differed significantly before and after the pandemic (χ^2^ = 12.50, df = 5, *p* = 0.029) (Table 3, Figure 3).

## 4. Discussion

This study employed TNA and topic modeling to examine the structure of scholarly discourse on unmet healthcare needs in patients with COPD. Six distinct topics were identified and mapped onto the five dimensions of healthcare access proposed by Penchansky and Thomas [11]: availability, accessibility, affordability, acceptability, and accommodation. The mapping confirmed that unmet healthcare needs were evident across all five dimensions rather than being confined to a single domain, underscoring the multidimensional nature of access barriers in COPD.

These findings suggest that unmet healthcare needs in patients with COPD constitute a multidimensional structural problem resulting from the interaction of access dimensions. Calverley [7] emphasized that COPD is a heterogeneous and complex disease with respect to pathophysiology, symptom presentation, and treatment response. This complexity makes it difficult to fully address patient needs through standardized approaches. Likewise, in a meta-analysis, Rahman et al. [26] found that unmet healthcare needs stem from constraints across multiple dimensions: affordability, accessibility, availability, and acceptability. They also reported that these effects are further exacerbated by socioeconomic disadvantages. In complex conditions such as COPD, the interactions among these dimensions may serve as key mechanisms that intensify structural inequities.

Addressing the unmet healthcare needs of patients with COPD therefore requires developing an integrated healthcare system that accounts for both the specific characteristics of the disease and the multidimensional nature of healthcare access. To this end, concrete strategies are necessary. These include expanding digital health infrastructure [27,28,29], stabilizing pharmaceutical supply and distribution systems [28,29], reducing regional disparities in healthcare resources [1,29], and establishing patient-tailored education and information delivery systems [2,26]. Supported by multidisciplinary collaboration and policy commitment, such interventions can reduce healthcare disparities and improve health outcomes [30,31].

A marked shift in topic distribution was observed before and after the COVID-19 pandemic. Prior to the pandemic, integrated care for advanced COPD (Topic 4) represented the largest share of the discourse. In the post-pandemic period, however, the relative prominence of guideline-based information and technology use (Topic 3) and availability and equity of COPD medications (Topic 6) increased. This shift reflects the contraction of in-person services and the redistribution of healthcare resources, which brought structural issues, such as digital information systems, mobile health (mHealth), and equitable drug distribution, to the forefront. Recent studies likewise confirm that mHealth and telemedicine became central mechanisms for service coordination during the pandemic, thereby accelerating their institutionalization across diverse healthcare systems [27,28].

The post-pandemic increase in the proportion of Topic 6 reflects heightened scholarly attention to issues of medication availability and economic accessibility. According to the WHO [29], shortages of essential medicines, delays in distribution, and restricted access to care threaten continuity of treatment for chronic conditions, with disproportionately severe effects on vulnerable populations. In certain regions, treatment interruptions and the use of alternative therapies increased, while purchases of essential medicines declined, thereby exacerbating health inequalities [32,33]. Likewise, Ponce et al. [34] documented higher rates of medication shortages, delays in care, and financial burdens among patients with chronic diseases during the pandemic.

This shift can be attributed to the concentration of policy and research attention on urgent priorities during the pandemic, such as infection control, diagnostic access, and self-management [35,36,37]. The decline was also linked to the expansion of remote and technology-mediated care models, which temporarily reduced the visibility of in-person, interaction-intensive palliative care.

Keyword network analysis further highlighted management, diagnosis, symptom, exacerbation, palliative care, pulmonary rehabilitation, and hospitalization as central domains, all showing high degree, betweenness, and closeness centralities. Together, these centrality patterns indicate that the terms function as key hubs and bridges structuring the scholarly discourse on unmet healthcare needs in COPD. They represent the clinical and care-related core of the literature, encompassing symptom control, exacerbation prevention, hospitalization management, rehabilitation, palliative care, diagnosis, and overall disease management [7,8]. As highly central nodes, these concepts should be prioritized in future research agendas and policy interventions.

This study has several limitations. First, when comparing the pre- and post-pandemic periods, the two phases were temporally asymmetrical: the pre-pandemic period spanned several decades, whereas the post-pandemic period covered only approximately 5 years. This imbalance may confound comparisons of topic proportions across periods. In addition, pandemic-related terminology may have appeared disproportionately in post-2020 studies due to indexing and publication biases, rather than reflecting genuine shifts in research priorities. Second, because eligibility was determined based on abstracts, misclassification may have occurred in judging whether a study truly reflected the patient perspective. Reliance on abstracts rather than full texts may also have limited the contextual details available for analysis. As a result, keyword extraction and topic modeling may have overemphasized highly visible themes in abstracts while underrepresenting nuanced or less frequently reported subtopics discussed in full texts, which should be considered when interpreting topic salience and differences across periods. Third, studies addressing provider or policy perspectives were excluded. These perspectives remain important for understanding unmet healthcare needs. Consequently, this exclusion may have reduced the comprehensiveness of the findings. Finally, only English-language abstracts indexed in international databases were included, which may have resulted in an overrepresentation of studies from high-income countries. These limitations call for cautious interpretation of the findings. Future research should incorporate full-text analyses, broader stakeholder perspectives, and more diverse sources to provide a more comprehensive and balanced understanding of unmet healthcare needs in COPD.

## 5. Conclusions

This study identified six major topics of unmet healthcare needs in COPD, each linked to multiple dimensions of healthcare access. The findings demonstrate that these needs are inherently multidimensional, encompassing issues of availability, accessibility, affordability, acceptability, and accommodation. A marked shift was observed after the COVID-19 pandemic, with scholarly attention moving from integrated end-of-life care toward guideline-based information delivery, technology utilization, and equitable pharmacotherapy. Keyword network analysis also revealed that symptom management, exacerbation, hospitalization, palliative care, diagnosis, and pulmonary rehabilitation constitute central domains within the discourse. Addressing these gaps through integrated, multidisciplinary, and policy-driven strategies is essential for reducing healthcare disparities and improving outcomes for patients with COPD. These findings may support evidence-informed priority settings to address unmet needs and related disparities in COPD, particularly in the post-COVID-19 context.

## Figures and Tables

**Table 1:** Network centrality metrics for core keywords.

Keyword	Frequency	Degree Centrality	Betweenness Centrality	Closeness Centrality
Management	133	0.172	0.370	0.305
Diagnosis	114	0.069	0.296	0.305
Symptom	111	0.172	0.700	0.377
Exacerbation	110	0.138	0.628	0.367
Palliative care	102	0.138	0.315	0.305
PR	91	0.069	0.133	0.244
Hospitalization	80	0.069	0.246	0.293
Smoking	80	0.035	0.000	0.203
Education	76	0.069	0.069	0.200
HRQoL	75	0.069	0.069	0.282
Medication	74	0.035	0.000	0.166
Primary care	70	0.035	0.000	0.203
Physician	70	0.035	0.000	0.172
Guideline	68	0.069	0.069	0.206
Information	65	0.035	0.000	0.276
Support	65	0.069	0.069	0.240
Spirometry	63	0.138	0.259	0.252
Resource	62	0.035	0.000	0.236
Cost	55	0.103	0.197	0.240
Quality	51	0.035	0.000	0.221
Approach	50	0.035	0.000	0.236
Area	50	0.035	0.000	0.236
Community	50	0.035	0.000	0.195
SES	49	0.035	0.000	0.168
Healthcare system	47	0.035	0.000	0.195
Availability	46	0.069	0.069	0.197
Knowledge	45	0.035	0.000	0.236
Severity	43	0.035	0.000	0.271
Caregiver	43	0.069	0.069	0.240
Family	43	0.035	0.000	0.195

HRQoL, health-related quality of life; SES, socioeconomic status; PR, pulmonary rehabilitation.

**Table 2:** Topics of unmet healthcare needs in COPD with corresponding healthcare access dimensions (5A framework).

	Title	Top Keywords (Probability)	Healthcare Access
Topic 1	Socioeconomic Inequalities in COPD Outcomes	SES (0.044) hospitalization (0.039) primary care (0.012) residence (0.009) predictor (0.009) insurance (0.008) smoking (0.008)	Availability Accessibility Affordability
Topic 2	Early Diagnosis and Symptom Management	exacerbation (0.059) diagnosis (0.040) symptom (0.032) smoking (0.030) spirometry (0.030) guideline (0.020) primary care (0.012)	Availability Acceptability
Topic 3	Guideline-Based Information and Technology Use	management (0.023) physician (0.021) guideline (0.015) adherence (0.013) information (0.012) mobile Health (0.012) technology (0.011)	Acceptability Accommodation
Topic 4	Integrated Care for Advanced COPD	palliative care (0.063) caregiver (0.022) symptom (0.016) support (0.015) HRQoL (0.014) management (0.013) breathlessness (0.011)	Availability Accommodation Acceptability
Topic 5	Improving Access to Pulmonary Rehabilitation	PR (0.162) referral (0.020) exacerbation (0.017) cost (0.016) exercise (0.013) hospitalization (0.013) benefit (0.012)	Availability Accessibility Accommodation Affordability
Topic 6	Availability and Equity of COPD Medications	medication (0.048) availability (0.027) medicine (0.026) LMICs (0.012) region (0.011) affordability (0.011) cost (0.010)	Availability Affordability

HRQoL, health-related quality of life; SES, socioeconomic status; PR, pulmonary rehabilitation; LMICs, low- and middle-income countries.

**Table 3:** Distribution of topics related to unmet healthcare needs in COPD before and after the COVID-19 pandemic.

	Pre-Pandemic n (%)	Post-Pandemic n (%)	Total n (%)	χ^2^ (*p*)
Topic 1	40 (15.7)	25 (12.8)	65 (15.3)	12.50 (0.029)
Topic 2	50 (19.6)	42 (21.4)	92 (21.4)	
Topic 3	34 (13.3)	38 (19.4)	72 (14.7)	
Topic 4	82 (32.2)	40 (20.4)	122 (28.2)	
Topic 5	32 (12.6)	28 (14.3)	60 (11.6)	
Topic 6	17 (6.7)	23 (11.7)	40 (8.8)	

## Data Availability

Dataset available on request from the authors.

## Supplementary Materials

The following supporting information can be downloaded at: https://www.mdpi.com/article/10.3390/healthcare14010082/s1, File S1: Dataset. Preprocessed bibliographic metadata and dominant topic assignments for included studies; File S2: PRISMA 2020 Checklist.

## Abbreviations

UHC	Universal Health Coverage
COPD	Chronic Obstructive Pulmonary Disease
5A	Availability, Accessibility, Affordability, Acceptability, Accommodation (Penchansky & Thomas model)
COVID-19	Coronavirus Disease 2019
TNA	Text Network Analysis
PubMed	(Proper noun—U.S. National Library of Medicine database)
Embase	(Proper noun—Elsevier biomedical database)
CINAHL	Cumulative Index to Nursing and Allied Health Literature
MeSH	Medical Subject Headings
PRISMA	Preferred Reporting Items for Systematic Reviews and Meta-Analyses
NetMiner	(Software name—not expanded abbreviation)
PFNet	PathFinder Network
LDA	Latent Dirichlet Allocation
c_v	Coherence metric for topic modeling (not acronym-based)
UMass	University of Massachusetts coherence metric
SPSS	Statistical Package for the Social Sciences
IBM	International Business Machines
IRB	Institutional Review Board

## Appendix A

### Appendix A.1. Full Search Strategies

**PubMed (searched 12 March 2025)**

(“Health Services Accessibility”[Mesh] OR “Healthcare Disparities”[Mesh] OR “Health Status Disparities”[Mesh] OR “Delayed Diagnosis”[Mesh] OR (“unmet healthcare need*”[tiab] OR “unmet health care need*”[tiab] OR “unmet medical need*”[tiab] OR “unmet treatment need*”[tiab] OR “healthcare gap*”[tiab] OR “health care gap*”[tiab] OR “care gap*”[tiab] OR “healthcare shortage*”[tiab] OR “health care shortage*”[tiab] OR “healthcare barrier*”[tiab] OR “health care barrier*”[tiab])) AND (“Pulmonary Disease, Chronic Obstructive”[Mesh] OR “Chronic Obstructive Pulmonary Disease”[tiab] OR COPD[tiab] OR “Chronic Bronchitis”[Mesh] OR “Emphysema”[Mesh] OR “chronic airflow obstruction”[tiab] OR COAD[tiab] OR COLD[tiab])

**Embase (searched 13 March 2025)**

((‘unmet medical need’/exp OR ‘health care access’/exp OR ‘health care disparity’/exp OR ‘health care barrier’/exp) OR (‘unmet need’:ab,ti OR ‘unmet healthcare need’:ab,ti OR ‘unmet medical need’:ab,ti OR ‘unmet health need’:ab,ti OR ‘healthcare unmet need’:ab,ti OR ‘unaddressed health need’:ab,ti OR ‘unfulfilled health need’:ab,ti OR ‘unserved medical need’:ab,ti OR ‘unmet treatment need’:ab,ti OR ‘unsatisfied healthcare need’:ab,ti OR ‘healthcare gaps’:ab,ti OR ‘healthcare shortage’:ab,ti OR ‘healthcare service deficiency’:ab,ti OR ‘healthcare inadequacy’:ab,ti OR ‘healthcare accessibility’:ab,ti OR ‘medical underuse’:ab,ti)) AND (‘chronic obstructive pulmonary disease’/exp OR ‘chronic obstructive lung disease’:ab,ti OR ‘chronic bronchitis’:ab,ti OR ‘emphysema’:ab,ti OR ‘chronic airflow obstruction’:ab,ti OR ‘chronic respiratory disease’:ab,ti OR ‘copd’:ab,ti OR ‘coad’:ab,ti OR ‘cold’:ab,ti)

**CINAHL (searched 15 March 2025)**

AB (MH “Healthcare Disparities” or MH “Health Services Needs and Demand+” OR “Unmet Needs” OR “Unmet Healthcare Needs” OR “Unmet Medical Needs” OR “Unmet Health Needs” OR “Healthcare Unmet Needs” OR “Unaddressed Health Needs” OR “Unfulfilled Health Needs” OR “Unserved Medical Needs” OR “Unmet Treatment Needs” OR “Unsatisfied Healthcare Needs” OR “Healthcare Gaps” OR “Healthcare Shortage” OR “Healthcare Service Deficiency” OR “Healthcare Inadequacy”) AND AB (“Pulmonary Disease, Chronic Obstructive” OR “Chronic Obstructive Pulmonary Disease” OR “Chronic Obstructive Lung Disease” OR “Chronic Bronchitis” OR “Emphysema” OR “Chronic Airflow Obstruction” OR “Chronic Respiratory Disease” OR “COPD” OR “COAD”)

### Appendix A.2. Custom Dictionaries for Text Preprocessing

(a) Synonym Dictionary (examples)

Representative Term	Synonyms Consolidated
diagnosis	diagnostic, detection
therapy	treatment, intervention
medication	drug, pharmacotherapy
exacerbation	flare-up, acute worsening
guideline	protocol, recommendation

(b) Stopword Dictionary (examples)

study, patient, result, data, article, purpose, method, conclusion

(c) Compound-term Dictionary (examples)

Compound Term	Notes
pulmonary rehabilitation	standardized as single term
health-related quality of life	standardized as single term
primary care	standardized as single term
mobile health (mHealth)	standardized as single term
